# A cell type-specific expression atlas of small and total RNA in the heart after myocardial infarction

**DOI:** 10.1038/s41597-025-05061-1

**Published:** 2025-05-19

**Authors:** Ranjan Kumar Maji, Ariane Fischer, Eva-Maria Rogg, Melanie Möller, Gilles Gasparoni, Martin Simon, Stefanie Dimmeler, Marcel H. Schulz

**Affiliations:** 1 Institute for Cardiovascular Regeneration, Goethe-University; German Centre for Cardiovascular Research (DZHK), Partner site Rhine-Main, 60590 Frankfurt, Germany; 2 Institute for Computational Genomic Medicine, Goethe University Frankfurt, 60590 Frankfurt, Germany; 3 Molecular Cell Biology & Microbiology, Wuppertal University, Wuppertal, Germany; 4 Epigenetics, Saarland University, 66123 Saarbrücken, Germany; 5 Present Address: Immundiagnostik AG, 64625 Bensheim, Germany

**Keywords:** Small RNAs, Data integration

## Abstract

Acute myocardial infarction (AMI) is a leading cause of mortality worldwide. MicroRNAs (miRNAs), among other small non-coding RNAs, shape the transcriptome and control cellular functions. Although single-cell technologies are now available to study myocardial ischemia response, the study of small RNA regulation is limited by depth of expression, capture efficiency and lack of full coverage of transcripts. In addition, the kinetic expression of miRNAs is unknown. Using paired small and total RNA sequencing, we built an expression atlas to study the temporal dynamics of miRNAs and genes in four major heart cell types after AMI. Expression dynamics reveal enriched functions highlighting cell type-specific AMI stress responses. Many deregulated mouse genes after AMI overlap with known human cardiovascular disease genes. The dataset is highly valuable for additional research on small and long non-coding RNAs, such as regulation of RNA variants by splicing or alternative ORFs. All in all, the RNA expression atlas provides a useful resource to study different roles of RNAs in major cell types of the heart after AMI.

## Background & Summary

Cardiovascular diseases are the major cause of death in the western world^1^. This is partly attributed to the consequences of myocardial infarction (MI), which can lead to ischemic heart failure. MI induces tissue ischemia resulting in acute cardiomyocyte (CM) death followed by an integrated reparative repair response mediated by a coordinated action of endothelial cells (EC), fibroblasts (FB) and hematopoietic cells (HC). To adapt to tissue ischemia, EC undergoes a transient switch to glycolysis and endothelial mesenchymal transition followed by a pro-angiogenic response which results in the re-growth of capillaries within the first two weeks after infarction^2^. Fibroblasts respond to injury by activating the inflammatory response through the secretion of cytokines and chemokines followed by matrix synthesis^3–5^. Furthermore, hematopoietic cells, mainly including short-lived neutrophils and monocytes are rapidly recruited after MI and are required for removal of cellular debris^6^.

Non-coding RNAs, specifically microRNAs (miRNAs), play a critical role in cardiac and vascular protection and postischemic heart failure by controlling mRNA stability or translation of their target genes^7–9^. However, the role of miRNAs and their function during postischemic myocardial repair in different cell types are not fully understood. It remains unclear how miRNA expressions are affected in each cell type after stress, which would further enable analyses on their functions.

In this study, we generated paired small RNA-seq and ribo-depleted total RNA-seq profiles at six timepoints after AMI stress induction in four major cell types of the murine heart, namely, cardiomyocytes, endothelial cells, fibroblasts and CD45-positive hematopoietic cells. Our data enables recapitulation of miRNAs as important regulators of AMI stress-related functions. The study presents a time-resolved expression atlas as a resource for further investigation of cell type-specific response after myocardial infarction.

## Methods and Results

### Ethics statement

All animal experiments were per institutional guidelines and approval by the ethics committee was granted for the project under title “Regulatorische RNA Netzwerke” ID: V 54 – 19 c 20/15 FU/1218 (04.10.2018 – 03.10.2023).

### Experimental setup

**Fig. 1:**
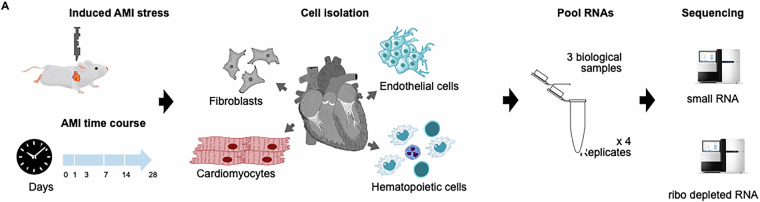
Experimental set up for the construction of the AMI expression atlas. (**A**) Experimental setup to analyse the effects of small RNAs after induced AMI stress, and sample collection at 6 timepoints (days): D0, D1, D3, D7, D14, D28; The selected timepoints covered periods of early AMI response (D0-D1-D3), recovery (D7-D14) and adaptation (D14-D28). After isolation of cardiomyocytes (CMs), endothelial cells (ECs), fibroblasts (FBs) and hematopoietic cells (HCs), small RNA and ribo-depleted total RNA sequencing was performed on RNA pooled from three mice for each biological replicate. Four technical replicates were created per time point for each cell type. D0 served as control.

To study the regulatory dynamics after myocardial infarction, we collected cell-sorted samples for each of the four major cell types: cardiomyocytes (CMs), endothelial cells (ECs), fibroblasts (FBs) and the hematopoietic cells (HCs) at six timepoints (Fig. 1). We conducted small RNA-seq for discovery of miRNAs and other small regulatory RNAs. In addition, we performed ribo-depleted total RNA-seq to measure the complete long RNA transcriptome without bias to poly-adenylated RNAs and coverage of non-coding RNAs (ncRNAs). The experimental setup thus revealed expression dynamics of genes and miRNAs in all four major cell types of the heart upon post-AMI stress.

The following steps were adhered to before the samples were sent for RNA library preparation (details in the section ‘RNA library preparation’):

#### Surgery

72 mice were divided into 8 groups - a control group, which was not operated on, and seven surgical groups, which were each operated on different days after MI was induced.

#### Harvest

2–4 animals were sacrificed per day and cell types were isolated. Each surgical group was assigned to each time point after AMI, i.e. D0, D1, D3, D7, D14 and D28. A maximum of 4 animals per time point were allowed to come from the same surgery and each time point consisted of animals from at least 3 different days of surgery, to minimize chances of batch effect (details in the section ‘Myocardial infarction and heart digestion’).

#### Cell type isolation

The cell types - cardiomyocytes, endothelial cells, fibroblasts and hematopoietic cells, were isolated from the hearts removed in step II. As these protocols are labor intensive, it was only possible to isolate cells from a maximum of 4 hearts per day (details in the section ‘Cardiac cell isolation’).

#### RNA isolation

RNA isolation was performed with 12 Qiagen kits. All buffers, including water, were pooled beforehand. The columns were all from the same batch. Isolation was always performed by the same operator, at the same location and with the same instruments. The isolated DNA was stored immediately at −80 °C. Since not all samples could be isolated in one run, the isolated cells were divided into 21 (3 × 7) groups. To reduce possible undesired effects of grouping, the division was carried out under the following conditions: first each RNA isolation group had to contain samples from each experimental time point (control, AMI D1, D3, D7, D14, D28). Second, each of these time points had to be composed of different cell types (each cell type could only occur once per time point of an isolation group). Third, the different cell types in an isolation group were not allowed from the same animal. The individual cell types are represented equally per isolation group. The amount of RNA was measured with Nanodrop.

#### Pooling

The amount of RNA obtained from the individual samples was not sufficient to enable deep sequencing. Therefore, the samples were pooled at the RNA level. This strategy had the additional advantage that the relative abundances found from sequencing could be validated with the RNA abundances from the remaining RNA of each sample not used for pooling. There were 12 RNA samples per time point and per cell type. These samples were pooled into 4 pools of 3 (2 if one sample was missing) samples per time point and cell type, such that the samples do not come from the same RNA isolation group. For each time point, the 4 pools of a cell type were collected from the same animals, to capture the physiological effects from the matched animal, and cross-talk between each cell type.

C57Bl6/J strain male mice (inbred) were used to ensure consistency of genetic characteristics and all surgeries were performed in a similar way. Same expert individuals performed all cell isolation and RNA isolations throughout the experimental duration. The experiments were conducted at one location, utilizing identical equipment and materials, with particular emphasis on excluding enzymes from diverse batches. This way we maintained stringent standards throughout the experimental process. The surgery, cell isolation and RNA isolation dates were considered when selecting the pooling partners at the RNA level to exclude possible batch effects due to different days for experiments. We followed strictly standardized, well-established protocols and utilized highly experienced technicians to minimize possible sample variabilities.

We focused on the early time points, as they are when the major changes in cell death (such as in cardiomyocytes), hypoxia response (such as in endothelial cells), and post-infarction inflammation occur, and the remodeling processes are initiated^10,11^. Investigating longer-term remodeling phases would also have been interesting, but it would have extended our resources.

### Myocardial infarction and heart digestion

Myocardial Infarction was performed in 12- to 14-week-old male C57Bl/6J-mice. Acute myocardial infarction (AMI) was induced by permanent ligation of the left anterior interventricular ramus of the descending coronary artery, under mechanical ventilation (as previously described in Rogg *et al*.^12^). Hearts were harvested from control mice without a MI (D0) as well as on day 1 (D1), day 3 (D3), day 7 (D7) and day 14 (D14) after MI. The digestion was performed by perfusion of a buffer containing collagenase type II. The digested heart tissue was carefully dissected into small pieces using forceps. Cells were then separated by carefully pipetting the cell suspension up and down. Digestion was stopped using Foetal Calf Serum. For each biological replicate RNA extracted from three mice was pooled to have sufficient amount of RNA available for sequencing. In total four biological replicates were created per time point and cell type.

### Cardiac cell isolation

Cardiomyocytes were enriched by performing repeated centrifugation steps (20 × g, 2 minutes, previously described in Rogg *et al*.^12^). The supernatant of the centrifugation step was used to isolate hematopoietic cells, endothelial cells, and fibroblasts. Hematopoietic and endothelial cells were isolated by magnetic separation (MACS cell separator, Miltenyi) with anti-CD45 magnetic beads (Miltenyi, # 130-052-301). The unbound, flow-through fraction was used for endothelial cell isolation utilizing Dynabeads® (Invitrogen by Life Technologies, Carlsbad, CA, USA) coated with anti-CD144 antibodies (BD Pharmingen, Allschwil, Switzerland, # 555289) by magnetic separation. Cells not bound to the magnetic beads or columns were used for cardiac fibroblast enrichment by a plating step with uncoated dishes. After 40 minutes, predominantly DDR2-positive fibroblasts adhered and were used for RNA isolation. The RNA amount obtained from the individual samples was insufficient to enable deep sequencing. Therefore, cells extracted from three mice were pooled to generate enough cells for one of four technical replicate sequencing samples for each timepoint.

### RNA library preparation

Total RNA was isolated from CM, EC, HC and FB using the Qiagen (Hilden, Germany) miRNeasy Kit involving column DNAse digestion. Small RNAs were converted into Illumina compatible libraries using the NEBNext small RNA library Kit from New England Biolabs (NEB), which involves 3′-ligation and 5′-ligation to small RNAs and therefore enriches for 5′-monophosphorylated and 3′-hydroxylated small RNAs, such as miRNA. To account for the exclusion of shorter RNA species and minimize the problem of small RNA size selection, the miRNeasy columns were prepared to counter-select RNAs below 10–15 nt. All four cell types had different RNA yields which depended distinctively on the cell type survivability response over time post-AMI. The variable response resulted in different amounts of cells in samples post-AMI induction and efficacy in cell sorting. This made it difficult to maintain the same RNA input volume for all cell types. This necessitated adjusting the PCR cycles for each cell type to account for the low RNA yield and attain enough volume for library preparation. However, all samples from each cell type were treated equally, thereby avoiding any cross-cell type bias. Due to the varying RNA amounts available for each cell type, we used the following parameters for input of total RNA and PCR cycles: CM-500ng 14 cycles, EC-50ng 16 cycles, HC-150ng 15 cycles, FB-150ng 15 cycles. After library amplification, we cut each library from 10% TBE (Tris-Borate-EDTA) PAGE gels, extracting the library size of approximately 140–150 bp which corresponds to 20–30nt miRNAs. The size exclusion of the final library by TBE gels cleavage ensured that the corresponding band of the miRNA were cut and the smear below the shorter RNA (most likely a result of RNA degradation product) were discarded for small RNA size selection. Libraries were then quantified using the Qubit dsDNA HS Assay (Thermo Fisher, Germany). Long RNA was converted into Illumina compatible libraries using the CATS Total RNA-seq Kit v2 with rRNA depletion (Diagenode, Belgium). After the depletion, this procedure involves the fragmentation of RNA, 3 ´-polyadenylation and cDNA synthesis primed by oligo-T. Addition of a template switch oligo in combination with a suitable reverse transcriptase, allows then the generation of double stranded cDNA with adaptor sequences for further amplification. We used the following parameters for input of total RNA before rRNA depletion and PCR cycles: CM-1000ng-10cycles, EC-500ng-13cycles, HC-200ng-13cycles, FB-200ng-13-cycles. We followed the manufacturers instructions for purification and size selection of the final libraries by AMPure beads (Beckmann, Germany) to enrich for approximately 300 bp products. Libraries were checked on Agilent Bioanalyzer DNA HS Kits and quantified using the Qubit dsDNA HS Assay (Thermo Fisher, Germany). All libraries, sRNA and long RNA, were single indexed, quantified by qPCR and cluster pooled accordingly. Illumina sequencing was carried out on a HiSeq. 2500 Platform HO mode. To avoid any cross-contamination, sequencing libraries were prepared in batches for each cell type with an index series of up to 48 indices (NEB catalog). This prevented any sample of a cell batch from having the same index. Other possible cross-contaminations were excluded by the batch preparation of samples.

### Ribo-depleted total RNA-seq analysis

Adaptor sequences were trimmed with TrimGalore [https://github.com/FelixKrueger/.

TrimGalore] based on the library preparation protocol. Salmon^13^ was used for the estimation of gene expression values using transcript alignments from STAR v2.28^14^.*--twopassmode Basic* option was used to map each sample separately to annotated junctions. Mouse annotation from GRCm38.87 with support level 5 was used for annotations of gene coordinates and splice junctions. All remaining parameters were kept unchanged.

### Small RNA-seq analysis

Small RNAs were quantified, following trimming (as in the previous section), using the small RNA-seq analysis pipeline from *nf-core*^15^ [https://nf-co.re/smrnaseq], where the full length of the small RNA reads were used for the pipeline. Reads less than 18 nts in length were filtered out. Normalised counts per million (CPM) estimates were obtained for mature miRNAs (canonical miRNA sequence and their hairpin sequence annotations from miRBase v21^16^).

## Data Records

Small RNA-seq and total RNA-seq data were deposited into the Gene Expression Omnibus database under accession number GSE252028^17^ Analyzed data and scripts to generate the figures are available on Zenodo under the 10.5281/zenodo.13694457^18^.

## Technical Validation

### RNA integrity

RNA quantity was fluorometrically measured using the Qubit 4 (Thermo Fisher, Germany) RNA HS Assay Kit and RNA integrity was verified using the Agilent Bioanalyzer RNA Pico chips.

### Cell type isolation

**Fig. 2:**
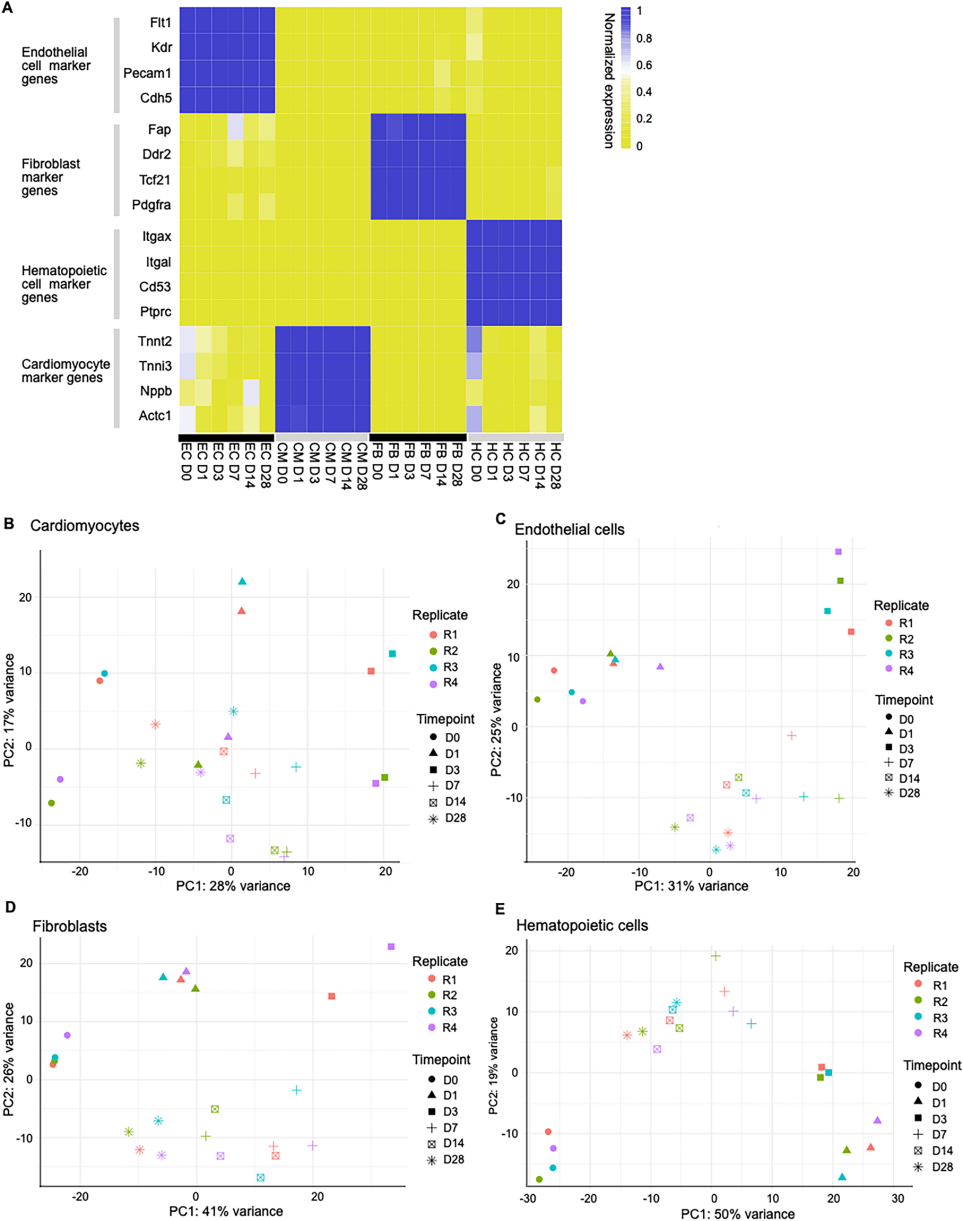
Cell type marker gene expression and sample distributions based on total RNA-seq data. (**A**) Marker gene expression for all cell types in all timepoints illustrate cell isolation efficiency. Gene expression was normalized per gene to range [0,1]. (**B**–**E**) Principal component analysis of gene expression value estimates with ribo-depleted total RNA-seq for samples after outlier removal in (**B**) CMs, (**C**) ECs, (**D**) FBs, and (**E**) HCs.

The efficiency of the isolation was verified using cell type-specific marker gene expression (Fig. 2A). These cell type-specific markers were used from previously reported markers in Rogg *et al*.^12^. The marker genes clearly showed higher normalized expression in their respective cell types compared to other cell types.

### Detection and removal of outlier samples

**Fig. 3:**
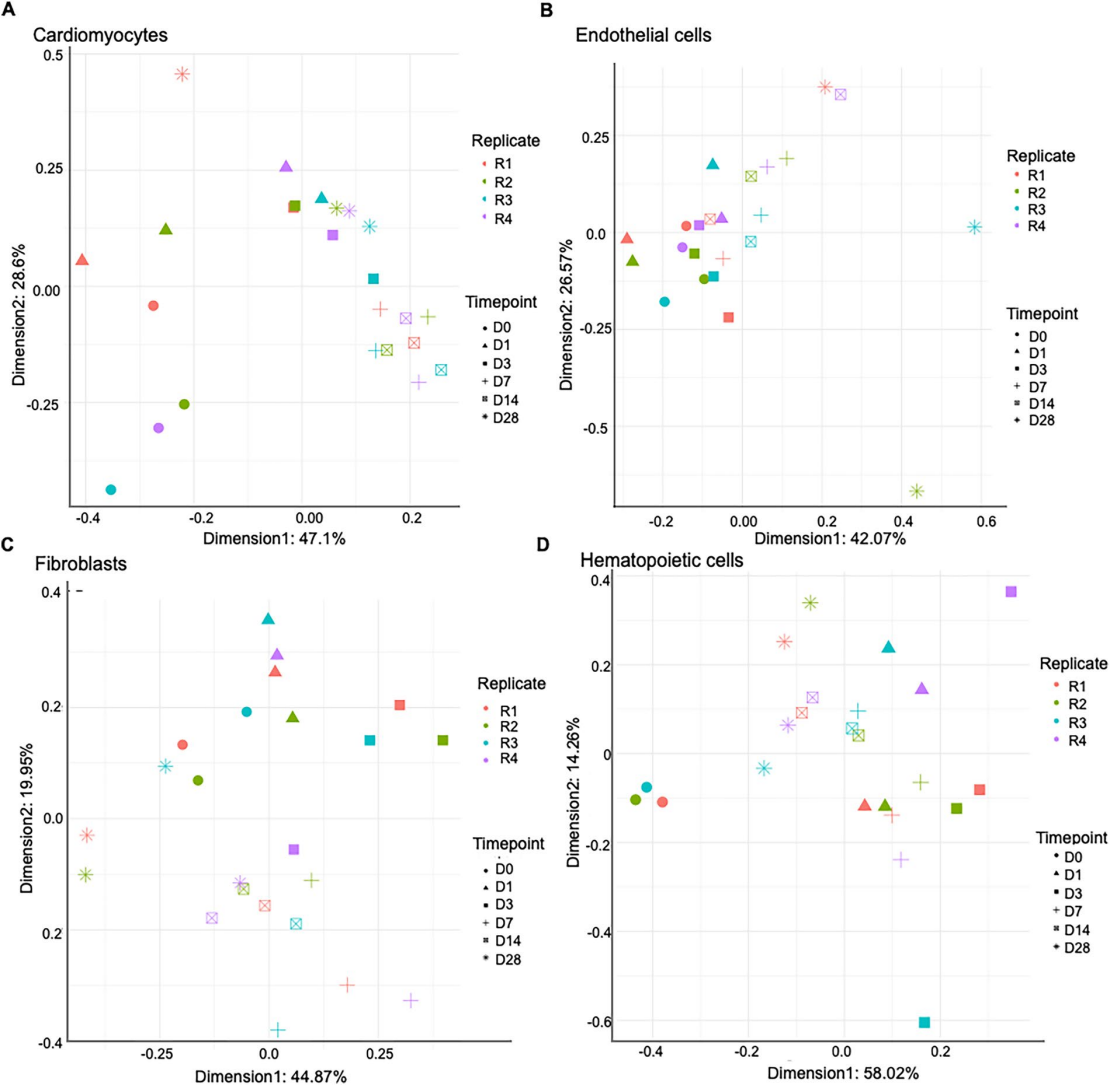
Sample distributions based on small RNA-seq. (**A**–**D**) Multidimensional scaling using the small RNA-seq in all four cell types *viz*. (**A**) CMs (**B**) ECs (**C**) FBs (**D**) HCs. This shows the variance of samples after outlier removal, as explained by small RNA-seq profiles.

For reliability in downstream analysis and to reduce sample variability, we removed the outlier samples. Outliers are the replicate samples with most variance within the sample group for a timepoint. Principal component analysis (PCA) was performed to detect outlier samples from total RNA-seq for all cell types. After PCA, replicate sample distributions were compared for each timepoint. Two replicates were removed as outliers from D3 in FBs and one from D28 in HCs, final plots shown in Fig. 2B–E. Multi-dimensional scaling (MDS, or Principal Coordinate Analysis), an alternative to PCA, is preferred for small RNA-seq samples due to a smaller number of features compared to RNA-seq [https://github.com/nf-core/smrnaseq]. After comparing small RNA-seq sample distribution around each time point using MDS, one replicate from D28 in ECs, one from D0 in HCs and one from D0 in FBs were removed. Final MDS analyses plots after outlier removal are shown in Fig. 3A–D.

### Verification of known candidates

To check the sanity of the total and small RNA-seq generated for all four isolated cell types after AMI, we investigate the expression dynamics of known genes and miRNAs either associated with cardio-specific mechanisms or enriched at certain time points following AMI in a cell type.

#### Expression dynamics of reported genes

**Fig. 4:**
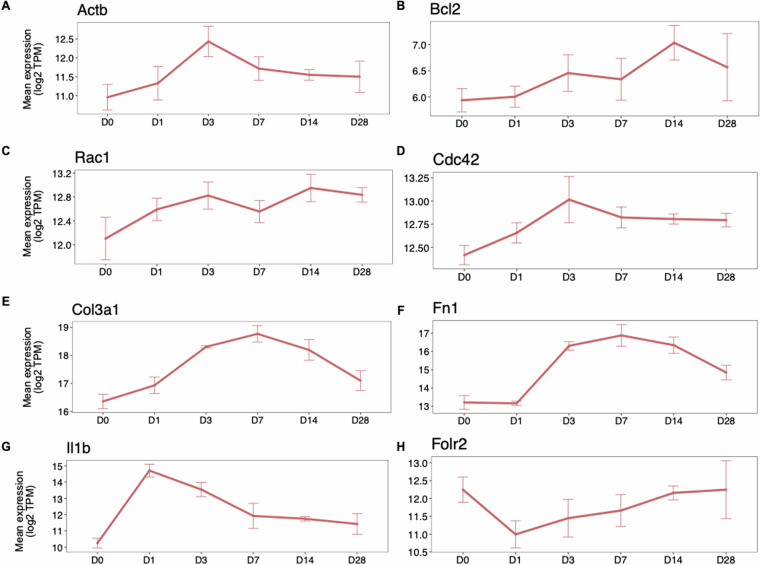
Gene expression dynamics post AMI. Gene expression dynamics (mean TPM over all replicates in each timepoint, log2) of (**A,B**) *Actb* and *Bcl2* in CMs; (**C,D**) *Rac1* and *Cdc42* in ECs, (**E,F**) *Col3a1* and *Rpl34* genes in FBs and (**G,H**) *Il1b* and *Folr2* in HCs at all timepoints after MI. The error bars show the variance over the replicates at each timepoint.

In cardiomyocytes, *Actb* (beta-actin or β-actin) has been found enriched at D3-D7 post-MI in heart tissue measurements and is known to play a role in cellular responses to stress^19^. Indeed, we found *Actb* to have the highest expression at D3 in CMs (Fig. 4A), the most abundant cell type in the heart. After myocardial infarction, there is an increase in β-actin dynamics in adult cardiomyocytes. This change is associated with localization of β-actin at Z-discs, costameres, and cell termini, improved contractility of cardiomyocytes and potential involvement in the hypertrophic response. In another example, anti-apoptotic proteins such as *Bcl2*, are known to remain highly expressed after MI^20^. We found that *Bcl2* showed a late response with increasing expression after MI induction (Fig. 4B)^21^.

Activation of RhoA signalling provides cardioprotection against stress^22^. *Rac1* and *Cdc42*, from the RhoA family, have been reported to respond to shear stress in endothelial cells and are involved in various functions, such as cytoskeletal dynamics, cell migration and angiogenesis after MI^23,24^. *Rac1* and *Cdc42*, showed upregulation in ECs (Fig. 4C,D) in our stress condition, but with noticeable differences in expression dynamics after D7.

Cardiac fibroblasts produce extracellular matrix (ECM) that provides mechanical support and transduces essential molecular signals. Following myocardial infarction, ECM changes dynamically and drives inflammation and repair^25^. Cardiac fibroblasts synthesize and secrete ECM proteins such as collagen (*Col3a1*, Fig. 4E) and fibronectin (*Fn1*, Fig. 4F). As expected, the expression of both genes increases from D1 as scar formation is part of the healing process in the injured heart. Then expression reduces after D7 when the acute repair phase is over.

Hematopoietic cells promote both cardiomyocyte death and inflammation as an immediate response, and facilitate the regeneration of damaged heart muscles post AMI^26^. After the wound is cleared from dead cells and matrix debris, endogenous inhibitory signals suppress IL-1 response resulting in inflammation repression and inflammatory infiltrate resolution^27^. We indeed found pro-inflammatory cytokine *Il1b* (Interleukin-1beta) response peaked expression at D1, with suppressed expression in the following timepoints (Fig. 4G). Macrophages are known to function as key regulators of tissue repair, regeneration and fibrosis and show immediate response to injury^28^. Upon cardiac injury, resident macrophage marker *Folr2* (folate receptor)^29^ gene expression reduced at D1 and gradually increased thereafter (Fig. 4H).

These various examples from all major heart cell types illustrate that the provided time-series resolution data not only support previous knowledge, but provides additional insight into early and late stress response and adaptation. Understanding the roles of these and other genes could provide insight into the development of new therapeutic strategies for improving cardiac recovery after infarction.

#### Expression dynamics of reported miRNAs

After AMI induction, each cell type showed distinct mature miRNA expression profiles. Most clinical studies have focused on functional and physiological effects of miRNA genes. miRNAs undergo differential processing, primarily in the event of stress^30^. This affects the biogenesis of mature miRNA arms (3p and 5p) and results in relative arm abundance differences along the AMI time course. Each mature arm is capable of repressing a distinct target gene repertoire^31^ and thereby regulating distinct functions. With small RNA-seq measurements at six timepoints after AMI in four cell types, we investigated mature arm expression dynamics of reported miRNAs in all cell types.

**Fig. 5:**
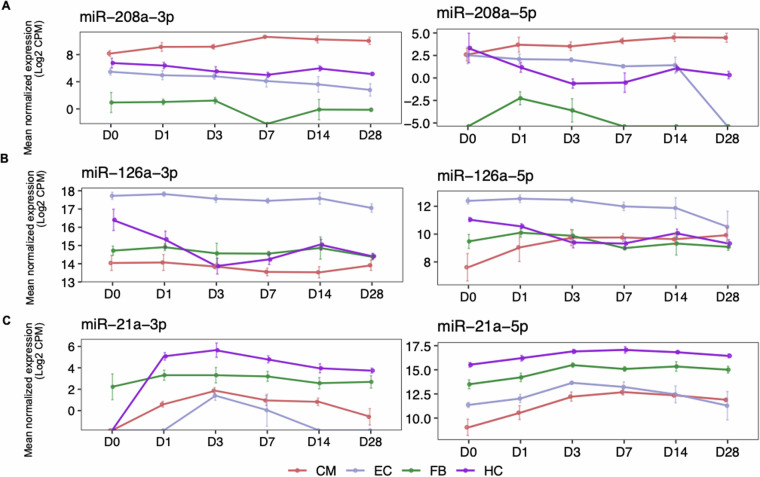
Expression dynamics of previously reported AMI-associated miRNAs among cell types. (**A**–**C**) Comparison of the expression dynamics (mean counts per million or CPM over all replicates at each time point) of mature arms of known cell type-specific miRNAs (**A**) miR-208a (**B**) miR-126a and (**C**) miR-21a in all cell types upon AMI. The error bars at each timepoint show the variance over the replicates.

miR-208, one of the early diagnostic markers of myocardial infarction, is essential for many myocardial functions and diseases^32^. Both arms of CM-specific miR-208a^33^, showed a sustained increase in expression upon MI (Fig. 5A) compared to other cell types. Another example of an endothelial-specific miRNA is miR-126, known to be a key player for endothelial function and integrity after MI^34^. Both of its mature arms showed a moderate decrease in expression over time in ECs. In CMs, however, the 3p arm showed a rapid decrease until D3 (Fig. 5B). miR-21, mostly reported in association with hematopoietic cells^35^, is known to have a significant increase in expression at day 1 and 3 in a myocardial infarct heart^36^. In HCs, indeed the 5p arm showed upregulation until D7 (Fig. 5C), while the 3p arm showed significant upregulation at D1 post AMI.

These analyses not only establish the quality of the dataset but also illustrate that the expression atlas can be used to investigate both arms derived from a miRNA gene, and compare their abundance between cell types. In addition, computational approaches that integrate paired small RNA and total RNA measurements enable investigation of small RNA regulatory dynamics and effects on gene regulation post AMI^37^.

## Data Availability

All scripts for the analysis used to generate the processed data and the visualizations in the manuscript are available on Zenodo under https://zenodo.org/records/14929916^18^.
